# Preservation of residual hearing after cochlear implant surgery: an exploration of residual hearing function in a group of recipients at cochlear implant units^☆^

**DOI:** 10.1016/j.bjorl.2018.02.006

**Published:** 2018-03-24

**Authors:** Katherine Gautschi-Mills, Katijah Khoza-Shangase, Dhanashree Pillay

**Affiliations:** University of the Witwatersrand, School of Human and Community Development, Department of Speech Pathology and Audiology, Johannesburg, South Africa

**Keywords:** Audiological, Cochlear implant, Preservation, Hearing, Surgical technique, Audiológico, Implante coclear, Preservação, Audição, Técnica cirúrgica

## Abstract

**Introduction:**

The preservation of residual hearing is becoming increasingly important in cochlear implant surgery. Conserving residual hearing is a positive prognostic indicator for improved hearing abilities.

**Objective:**

The primary aim of the study was to explore the preservation of residual hearing following cochlear implantation in a group of recipients at two major cochlear implant centers.

**Methods:**

A quantitative paradigm was adopted and exploratory research conducted within a retrospective data review design. The sample consisted of 50 surgical records and 53 audiological records from 60 observations (53 patients, seven of whom were implanted bilaterally). The records were selected using purposive sampling and consisted of records from participants ranging from six to 59 years of age. The average time of when the postoperative audiograms were performed in the current study was 24.7 months (s.d. = ±9.0). Data were analyzed through both qualitative and inferential statistics and a comparative analysis of unaided pre- and postoperative audiological test results was conducted.

**Results:**

Results indicated a high success rate of 92% preservation of residual hearing with half of the sample exhibiting complete preservation in cochlear implant recipients across all frequencies postoperatively. A total postoperative hearing loss was found in only 8% of cochlear implantees across all frequencies. There was no relationship between preoperative hearing thresholds and preservation of hearing postoperatively. The two main surgical techniques used in the current study were the contour on stylet and the advance off-stylet techniques, with the majority of surgeons utilizing a cochleostomy approach. From the findings, it became apparent that the majority of cases did not have any reported intraoperative complications. This is a positive prognostic indicator for the preservation of residual hearing.

**Conclusion:**

Findings suggest improved cochlear implant surgical outcomes when compared to previous studies implying progress in surgical techniques. The surgical skill and experience of the surgeon are evidenced by the minimal intraoperative complications and the high success rate of hearing preservation. This is a positive prognostic indicator for individuals with preoperative residual hearing as the preserved residual hearing allows for the potential of electro-acoustic stimulation, which in turn has its own hearing benefits.

## Resumo

**Introdução:**

A preservação da audição residual tem se tornado cada vez mais importante na cirurgia de implante coclear. Conservar a audição residual é um indicador prognóstico positivo para melhorar as habilidades auditivas.

**Objetivo:**

Avaliar a preservação da audição residual pós-implante coclear em um grupo de pacientes de dois grandes centros de implantes cocleares.

**Método:**

Foram adotados um paradigma quantitativo e uma pesquisa exploratória em um projeto retrospectivo de revisão de dados. A amostra consistiu de 50 registros cirúrgicos e 53 registros audiológicos de 60 observações (53 pacientes, sete dos quais foram implantados bilateralmente). Os registros foram selecionados com amostragem proposital e consistiram em registros de participantes de seis a 59 anos. Neste estudo, o tempo médio para audiogramas no pós-operatório foi de 24,7 meses (dp = ± 9,0). Os dados foram analisados através de estatísticas qualitativas e inferenciais, foi feita uma análise comparativa de resultados de testes audiológicos pré e pós-operatórios, sem auxílio de aparelho auditivo.

**Resultados:**

Os resultados indicaram uma alta taxa de sucesso, de 92%, de preservação da audição residual, metade da amostra dos implantados exibia preservação completa em todas as frequências no pós-operatório. Perda auditiva total em todas as frequências no pós-operatório foi observada em apenas 8% dos implantes cocleares. Não houve relação entre limiares auditivos pré-operatórios e preservação auditiva no pós-operatório. As duas principais técnicas cirúrgicas usadas no presente estudo foram *contour on stylet* e a técnica *advance off-stylet* e a maioria dos cirurgiões usou uma abordagem por cocleostomia. A partir dos achados, tornou-se evidente que a maioria dos casos não apresentou complicação intraoperatória. Esse é um indicador prognóstico positivo para a preservação da audição residual.

**Conclusão:**

Os achados sugerem melhoria dos resultados cirúrgicos do implante coclear quando comparados com estudos prévios, sugerem um progresso nas técnicas cirúrgicas. A habilidade cirúrgica e a experiência do cirurgião são evidenciadas pelas complicações intraoperatórias mínimas e pela alta taxa de sucesso de preservação auditiva. Esse é um indicador prognóstico positivo para indivíduos com audição residual pré-operatória, uma vez que a audição residual preservada possibilita o potencial de estimulação eletroacústica (EAS – do inglês *Electric Acoustic Stimulation*), que, por sua vez, tem benefícios auditivos próprios.

## Introduction

Cochlear implants have revolutionized the manner in which rehabilitation of patients with severe to profound hearing impairment in restoring speech understanding is approached.^1^ Success in conservation of residual hearing after cochlear implantation has benefited patients with high levels of residual low frequency hearing who were not previously considered for conventional cochlear implantation.^1^

Verhaegen et al.^2^ have discussed how the advent of cochlear implantation has enabled individuals with bilateral profound sensori-neural hearing impairment (who obtain minimal or no benefit from hearing aids) to be effectively rehabilitated. Gstoettner et al.^3^ have observed that over the last two decades, cochlear implants have become the standard mode of intervention for individuals with profound sensori-neural hearing loss. In their study in 2009, Lenarz et al.^4^ held that cochlear implantation is the “current treatment of choice in patients with profound sensori-neural hearing loss” (p. 22).

In the past, only profoundly hearing-impaired individuals with no residual hearing were implanted,^2^ with those with residual hearing in low frequencies excluded due to the intraoperative risks resulting in loss of residual hearing.^3^, ^5^ Within the last decade, the inclusion criteria for recipients have, however, been expanded to include individuals with some residual hearing.^2^, ^3^ This expansion in inclusion criteria has been due to technological advancements,^6^ improvements in surgical techniques^3^, ^4^ and less traumatic electrode array insertion.^4^ Improved outcomes have allowed these patients to experience better performance post-surgery, with cochlear implants becoming the main form of management for severe to profound bilateral hearing loss.^5^, ^7^, ^8^

A review of earlier literature on traditional measures to ensure hearing preservation makes comparisons of studies complex, and indicates that caution should be taken when comparing findings as is the case with the current study where retrospective record reviews were conducted without any active manipulation of variables. As early as 1997 it was reported in a study conducted by Hodges et al.^9^ that there was preservation of residual hearing in about 50% of implantees. However, early reports in the literature on the efficacy of cochlear implantation indicated that there were significant decreases in residual hearing following surgery, with most implantees losing their residual hearing following implantation.^8^ As a result, only profoundly hearing-impaired individuals were implanted.^8^, ^10^ Adunka et al.^11^ reported on the majority of cochlear implant recipients in their study retaining their residual hearing postoperatively. However, 90% achieved only partial preservation, leading them to conclude that additional effort is required to enhance surgical protocols with the goal to achieve total hearing preservation in all cases.

Several authors^2^, ^7^, ^12^, ^13^ have argued for the importance of hearing preservation with cochlear implantation. Verhaegen et al.^2^ and Kiefer et al.^7^ argue that residual hearing is a good prognostic indicator for successful performance with cochlear implants. These authors have demonstrated that residual hearing following cochlear implant surgery allowed for long-lasting and stable performance related to speech understanding, as well as improved speech perception.

Preserved hearing has also become beneficial in one of the most significant advances, which is the introduction of bimodal Electric-Acoustic Stimulation (EAS). EAS involves the preserved low frequencies being amplified by means of acoustic stimulation in the form of hearing aids, while the high-frequency hearing loss is being addressed with electrical stimulation in the same ear by means of a cochlear implant.^2^ This is used for implantees whose low to mid-frequency residual hearing has been preserved postoperatively and has considerable benefits for hearing, hence the importance of studies such as the current one which aimed to explore the preservation of residual hearing function in a group of cochlear implant recipients.

### The following were the research objectives

The primary objective of the study was to explore the preservation of residual hearing function in a group of cochlear implant recipients by:

Describing and performing a comparative analysis of hearing function before and after cochlear implant surgery.
Determining to what extent residual hearing had been preserved or lost.
Establishing whether a relationship exists between the hearing findings and the surgical technique employed, as well as the incidence of intraoperative complications.

## Methods

### Research design

Within a quantitative paradigm, a retrospective data review design was adopted.^14^ The researcher retrospectively examined existing surgical records as well as pre- and postoperative unaided audiological testing results to determine whether residual hearing had been preserved. Due to the retrospective nature of the study, no manipulation of any variables was employed to influence the postoperative outcomes, and this included the type of surgical technique employed.

### Participant selection

#### Sampling strategy

Purposive sampling technique was used to recruit participants’ files.^14^

### Participant description

The records of participants included audiological testing data and surgical records from cochlear implantees with preoperative residual hearing. Participants consisted of male and female, unilateral and bilateral cochlear implant recipients from two cochlear implant units. The children had to be at least 6 years old (to increase reliability of pure tone test findings) at preoperative hearing testing and the adults not more than 59 years old (to minimize the influence of presbycusis) at postoperative testing. The average time of when the postoperative audiograms included in the current study were performed was 24.7 months (s.d. = ±9.0).

A pre-requisite of this study was that the participants had to have some preoperative residual hearing at any or all of the following frequencies: 125 Hz, 250 Hz, 500 Hz, 750 Hz, 1000 Hz, 1500 Hz, 2000 Hz, 3000 Hz, 4000 Hz, 6000 Hz and 8000 Hz.

### Participant sample size

The sample comprised 50 surgical records and 53 audiological participant records. Although there were 53 audiological participant files, seven of these individuals were bilaterally implanted, yielding a total sample size of 60 ear observations.

### Selection criteria

#### Participant inclusion criteria

The following are factors considered in the inclusion criteria:

Cochlear implants: The participants must have been fitted with a cochlear implant unilaterally or bilaterally at least one month prior to postoperative pure-tone testing.
Hearing status: The participants were required to have had a moderate sloping to profound, a severe to profound or a profound sensorineural hearing loss (that is, some residual hearing at any of the frequencies – not a corner audiogram) in both ears and must have had some degree of benefit, even if only minimal, from hearing aids.
Speech discrimination: Due to the fact that the participants underwent cochlear implantation, it was assumed that preoperatively, the participants scored ≤50% for sentence recognition in the ear to be implanted and ≤60% in the non-implanted or contralateral ear or bilaterally for speech discrimination, as this is a criterion for cochlear implantation.^15^
Auditory nerve functioning: Given the fact that the participants had cochlear implants, the researcher could make the assumption that the participants’ auditory nerve fibers were intact so that they could receive electrical stimuli inside the cochlea.^16^
Medical condition: It was assumed that the participants’ medical conditions and inner ear structures met the requirements for implantation, as these are criteria for candidacy.^16^
Age: One of the inclusion criteria was that the participants’ ages had to range between 6 and 59 years to ensure reliability of results and to exclude presbycusis loss.

### Data collection

#### Audiological data review

The following data was collected from each participant file: cochlear implant unit (Unit A/Unit B); left/right ear; Bilateral/unilateral implant; time (months) between last preoperative hearing test and surgery and time (months) between surgery and first postoperative hearing test.

The hearing thresholds (in dB) of the pure-tone air-conduction testing conducted preoperatively and postoperatively at the following frequencies: 125 Hz, 250 Hz, 500 Hz, 750 Hz, 1000 Hz, 1500 Hz, 2000 Hz, 3000 Hz, 4000 Hz, 6000 Hz and 8000 Hz.

The change: the post-pre difference between the hearing thresholds (in dB) of the pure-tone testing results, at the above frequencies where these frequencies could be measured.

The researcher collected additional data, which included the following: age, gender, etiology, duration of hearing loss prior to implantation (in years), implant type, implant manufacturer, electrode type, electrode array insertion, electrode array depth.

#### Surgical technique and Intraoperative complications

The types of cochlear implants, electrode array type, insertion depth and surgical technique used are summarized in Table 1.

**Table 1:** Implant type, electrode type, insertion depth and surgical technique.

*Implant type*	
NF – CI24RE	50%
CN – CI 512	25%
Other	17%
Missing	8%

*Electrode type*	
Contour advanced	83%
Other	8%
Missing	8%

*Insertion depth*	
Complete	93%
Partial	2%
Missing	5%

*Surgical technique*	
Contour on stylet	8%
AOS (advance off-stylet)	67%
Other	20%
Missing	5%

*n* = 60; number of participants, 53.

*Note*: NF-CI24RE, Nucleus Freedom cochlear implant with Contour Advance or Straight electrodes; CN-CI 512, Cochlear Nucleus 5 cochlear implant.

#### Cochlear implant type

Fifty percent of implantees were given the CI24RE implant and 25% the CI512.

The category “Other” (17%) included inter alia the cochlear implant type – Nucleus CI24R with Contour Advance electrode (*n* = 5).

#### Electrode array type

A large majority of the participants (83%) had the contour advance electrode array implanted.

#### Electrode array insertion depth

A total of 94% of the participants underwent complete insertion of the electrode array. Very few patients (a mean of 2%) underwent partial insertion of the electrode array.

#### Surgical techniques

The two main surgical techniques used in the current study were the contour on stylet and the AOS (advance off-stylet) technique with the AOS technique being the most used (67%).

The surgical technique used did not have a negative effect on the postoperative hearing threshold levels. The use of the AOS or contour on stylet or “Other” technique did not change the postoperative outcome in terms of preservation of residual hearing.

The majority of surgeons utilized a cochleostomy approach, with the exception of four cases where the round window approach was used.

### Data analysis

The researcher chose to adopt James et al.’s ^17^ approach whereby an artificial value is used to represent the total loss of residual hearing. James et al.^17^ suggested that this could occur at the maximum output of the audiometer.

The researcher chose to assign an artificial or numerical value to represent the total loss of residual hearing at the maximum output of the audiometer plus 5 dB. The researcher also elected to represent the maximum output of the audiometer as 120 dB.

In calculating the preoperative and postoperative hearing threshold levels, when the hearing threshold level was “no response” (NR), the researcher assigned a value of 125 dB to NR. This value of 125 dB was assigned to replace NR in order to determine the residual hearing or lack thereof pre- or postoperatively.

### Statistical procedures

Data analysis was carried out in STATISTICA (version 10). The results were analyzed using descriptive and inferential statistics.^14^ For inferential statistics, the 95% confidence level was used throughout, unless otherwise specified.

### Reliability and validity

To minimize participant variables from impacting on the reliability and validity of results, the researcher excluded any records where participants were reported to have been “unreliable” during testing or where there was “questionable reliability”. Further, as in Kiefer et al.’s^7^ study, any “vibrotactile” responses reported by the participants were excluded from calculations. The researcher worked on the assumption that the audiologists would have reported any unreliable responses, and would have adhered to best practice in terms of test protocols, sound proofing, as well as equipment calibration.

### Ethical considerations

Prior to the commencement of the study, the researcher obtained ethical clearance from the University's Human Research Ethics Committee (Medical) (Protocol n° M111037). Thereafter, the researcher obtained permission from all the relevant authorities. Furthermore, the work adhered to the Helsinki Declaration of 1975, as revised in 2008.

## Results and discussion

### Demographic profile

Table 2 represents the demographic profile of the sample in the current study.

**Table 2:** Demographic profile of observations (*n* = 60).

Variable	Overall
Number of participants	53
Number of bilateral implants	7
Ears (% of total ears)	60
Ears (% of L ears)	40%
Age at surgery (mean ± 95% Confidence Interval)	30.8 ± 3.6
Gender: % male	36%

As depicted in Table 2, a total of 60 observations formed the basis of the current study. The majority of the participants (64%) were female, with the mean age being 30.8 yrs. Seven participants were implanted bilaterally.

The sample size of the current study is believed to be adequate and had a greater number of observations than some other studies reported in the literature. Gstoettner et al.,^3^ for instance, conducted a highly successful clinical trial using the Med-El Flex EAS array, wherein the residual hearing of all the recipients was preserved postoperatively, with a sample size of nine recipients, limiting the generalization potential of their study. Lenarz et al.^4^ conducted a clinical trial with 24 participants (and 32 observations).

### Hearing function before surgery (PRE)

Data could only be recorded for 11 participants at 125 Hz. This is an important finding which requires attention by the audiology community within this context as Franks et al.^18^ assert that criteria for testing protocol must include 125 Hz for clinical testing. Furthermore, there was limited data for all inter-octaves. This was thought possibly to be due to the fact that according to Franks et al.,^18^ when conducting hearing testing for clinical purposes, half-octaves are only “sometimes” tested.

**Figure 1:**
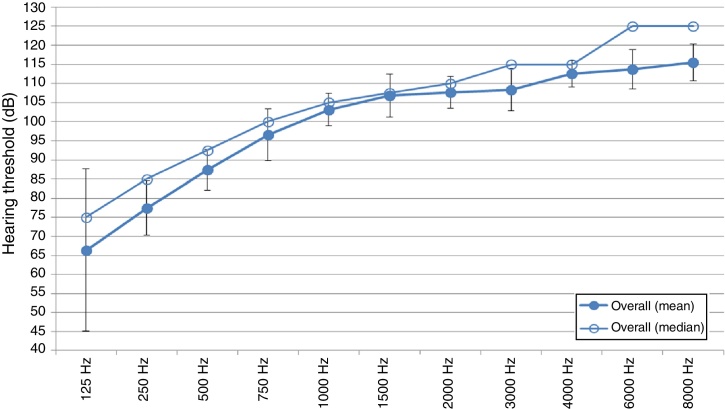
Preoperative hearing threshold level(s) overall (PRE HTL overall).

Fig. 1 depicts the preoperative hearing threshold levels at individual frequencies from 125 Hz to 8000 Hz for all the participants. Results indicated that preoperatively, participants in the overall sample presented with some degree of low frequency residual hearing with poorer hearing in the high frequencies. As the frequencies increased from 125 Hz to 8000 Hz, so the hearing loss increased. A General Linear Model (GLM) was used to confirm this audiogram configuration which showed more residual hearing in the low frequencies preoperatively. This was expected and can be explained by the tonotopicity of the cochlea, where high frequencies are more susceptible to damage than lower frequencies.^19^ The degree of the hearing loss pre-implant was generally in the severe to profound range as depicted in Fig. 1.

### Hearing function after surgery (POST)

**Figure 2:**
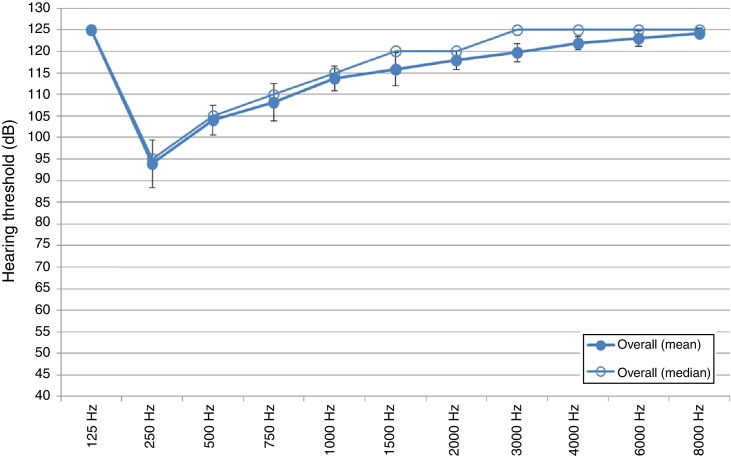
Postoperative hearing threshold levels overall (POST HTL overall).

As seen in Fig. 2, the results obtained indicated a high frequency loss postoperatively, with some preservation of the low frequencies and slight preservation of the mid-frequencies. At 8000 Hz there was a total loss of hearing, while at 250, Hz the hearing threshold level was 95 dB. Although the results indicated some preservation of low frequency and mid frequency hearing (the latter to a lesser degree), it was clear from the results that there was some loss in residual hearing indicated by hearing threshold levels dropping from before surgery to after surgery.

### Change in hearing function

**Figure 3:**
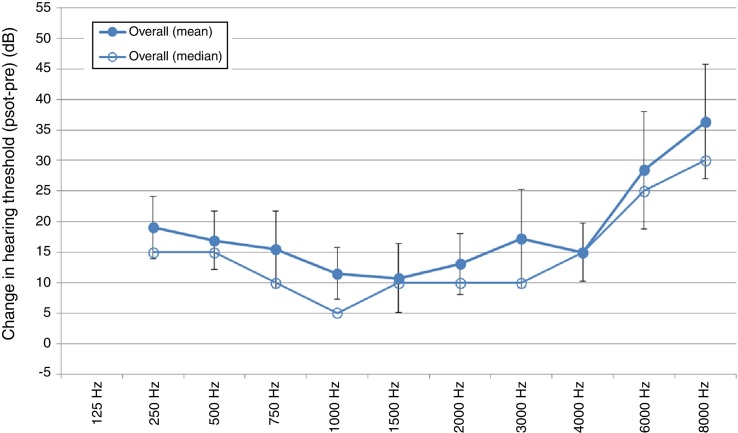
Change in hearing threshold level(s) overall (CH HTL overall).

Overall, as depicted in Fig. 3, there was a mean loss in residual hearing, particularly in the high frequencies. There was also a loss in the low and mid frequencies, but to a lesser degree than the high frequencies.

The change in residual hearing indicated by loss of hearing, which was less in the mid and low frequencies, is clinically significant. This preservation of hearing in the low and mid frequencies assists with speech perception in noise and allows for the consideration of EAS – acoustic amplification with a hearing aid and a cochlear implant for the high frequencies.

It was expected that there would be a decrease in hearing threshold levels in the high frequencies due to the placement and the insertion of the electrode in the cochlea and due to the tonotopicity of the cochlea. Additionally, the high frequencies are more susceptible to damage, given their placement in the cochlea, as found in the current study.

**Figure 4:**
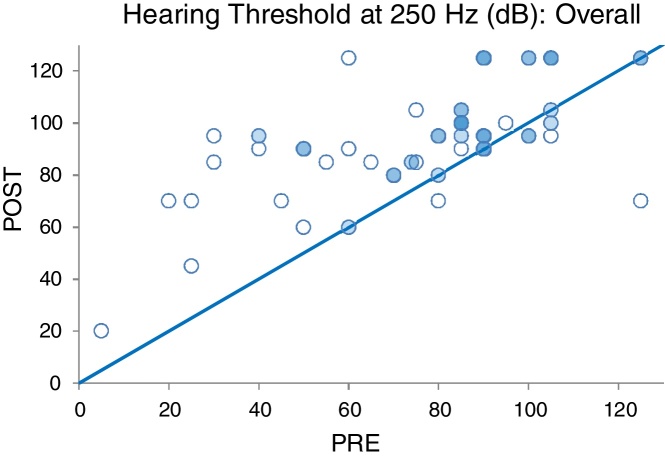
Pre- and postoperative hearing threshold levels at 250 Hz (PRE-POST HLT 250 Hz overall).

**Figure 5:**
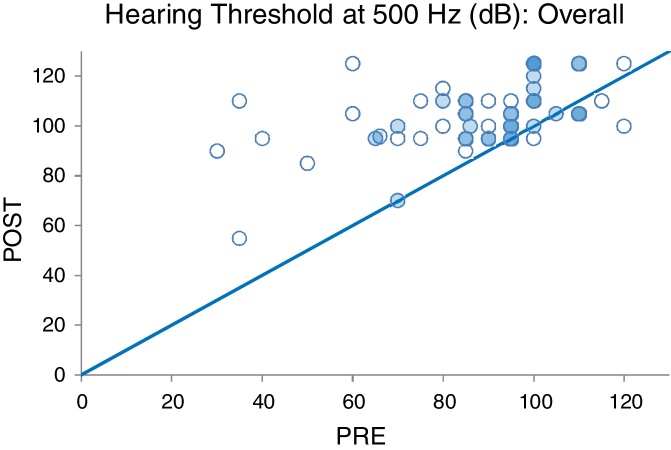
Pre- and postoperative hearing threshold levels at 500 Hz (PRE-POST HLT 500 Hz overall).

The preoperative and the postoperative hearing threshold levels were plotted on the same graph in order to better depict the change in residual hearing on unity lines as illustrated in Figure 4, Figure 5. The majority of cases lay above the unity line. This is indicative of hearing loss having taken place.

### Hearing preservation

#### Classification of change in hearing function

The change in hearing function from preoperative to postoperative hearing threshold levels was classified for each participant according to the scheme which is in line with that by Gstoettner et al.^3^ and Balkany et al.^5^ Additionally, the researcher chose to include all frequencies in this study so as to obtain more detailed information with regard to the change in residual hearing.

Three different combinations of frequencies were used as inputs for the above classification: Gstoettner et al.^3^: Average of 125–750 Hz; Balkany et al.^5^: Average of 250, 500 and 1000 Hz; All: Average across all frequencies.

The change in hearing function, according to the three classifications is provided in Table 3.

**Table 3:** Gstoettner et al.,^3^ Balkany et al.^5^ and Researcher's classifications.

Classifications	Gsteottner et al. classification	Balkany et al. classification	Researcher classification
Hearing preserved	53	55	55
	90%	92%	92%
Complete preservation	23	24	30
	39%	40%	50%
Partial preservation	30	31	25
	51%	52%	42%
Complete loss	6	5	5
	10%	8%	8%
Totals	59	60	60

**Figure 6:**
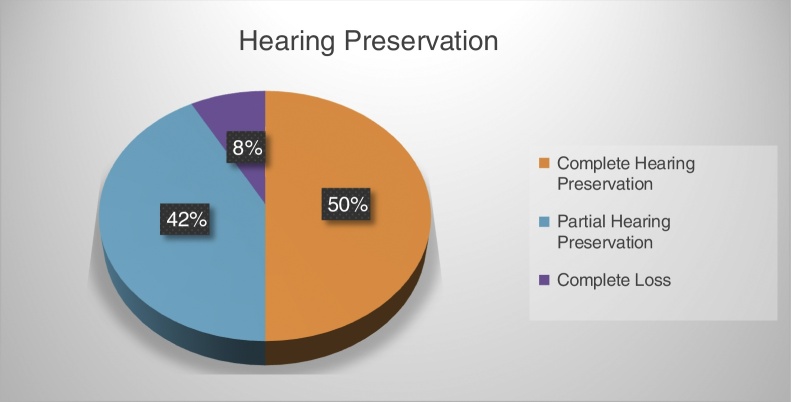
Percentage of hearing preservation in current sample (*n* = 60).

From Table 3, it is evident that the vast majority of the recipients preserved their hearing either partially or completely. There was no significant difference between the three classifications used. If one adopted the Balkany et al.^5^ classification, overall, 92% of the implantees experienced hearing preservation postoperatively – either partial or complete hearing preservation. When analyzed further, as depicted in Fig. 6, 50% experienced complete hearing preservation (0–10 dB change in hearing threshold levels) and 42% partial hearing preservation (>10 dB change in hearing thresholds). A small minority (8%) of the cochlear implantees experienced a total loss of residual hearing postoperatively as evidenced by absent responses at all frequencies postoperatively.

In summary, current findings indicate that the vast majority of the cochlear implantees experienced preservation of residual hearing – either partial or complete hearing preservation. These are positive findings that have implications for both clinical and surgical management of patients with cochlear implants.

#### Intraoperative complications

As depicted in Table 4, the main categories for intraoperative complications included adhesions (3%), drill out of basal turn (3%), Gusher (5%), and trauma (10%). A significant majority (68%) of case files indicated that there were no complications.

**Table 4:** Surgical complications in the current sample.

Intra-operative complications	
Adhesions	3%
Drill out of basal turn	3%
Gusher	5%
Trauma	10%
Other	3%
None reported	68%
Missing	7%

*n* = 60; number of participants, 53.

Hodges et al.’s^9^ study demonstrated that hearing was only preserved in 50% of implantees. In Di Nardo et al.’s^8^ study the majority of implantees retained their residual hearing. However, despite an atraumatic surgical approach being used, 22% experienced a total loss of hearing following surgery.

Balkany et al.^5^ reports on findings from the 1980s which indicated preservation of pure-tone threshold levels in approximately 33% of implantees postoperatively. In the 1990s conservation of residual hearing was seen in approximately half their patients post-surgery; with this figure increasing to a preservation rate of 89% postoperatively in 2006 using the Nucleus Freedom Contour Advance electrode and the advance off-stylet technique. Findings in the current study show a preservation rate of 92%, which implies a more successful hearing conservation outcome overall than in most previous studies. Furthermore, findings in the current study showed a higher rate of partial and complete hearing preservation according to the similar classification – that is, 50% and 42% for complete and partial preservation respectively. This improvement over the last decades can be attributed to a combination of advancements in surgical techniques and cochlear implant technology.^5^

Verhaegen et al.^2^ observed retention of residual hearing postoperatively in 70% of implantees in their study after utilizing a soft surgery technique with the Nucleus Contour electrode. Although these findings indicate an improvement over earlier studies, the successful overall preservation rate of 92% found in the current study was considerably higher.

The relationship between the postoperative hearing findings and degree of preoperative hearing loss was also explored in the current study. There was no significant correlation between the preoperative hearing threshold levels and the preservation of postoperative residual hearing (*p* = 0.174). The degree of preoperative hearing loss did not determine the outcome of the postoperative hearing threshold levels. These findings are consistent with those by Cosetti et al.^20^ who found that there was no significant relationship between the low-frequency pure-tone-average and the postoperative hearing findings. These findings are, however, different to those of Balkany et al.^5^ where a relationship between the degree of preoperative hearing loss and the hearing findings post operatively was found. In their study, patients who had a greater degree of preoperative hearing loss tended to experience a complete loss of residual hearing postoperatively.

Current findings showed commonalities with those of Roland et al.^21^ with regard to the surgical techniques, electrode insertion and the electrode arrays used. In the study of Roland et al.^21^ they examined the use of Contour Advance electrodes with the AOS technique. Atraumatic insertion was achieved by precise perimodiolar positioning in the scala tympani, thereby affording protection to intracochlear structures.^21^ This is consistent with current findings where the majority of surgeons utilized the AOS technique with results showing successful preservation of residual hearing in majority of cases.

The positive findings in the current study have demonstrated that cochleostomy approach has achieved success in preserving residual hearing postoperatively. Mangus et al.^22^ asserts that the round window approach has advantages for the conservation of residual hearing over the cochleostomy approach, which may result in intracochlear trauma. Briggs et al.^23^ observed in their study that both approaches successfully avoided trauma to the cochlea during surgery, thus conserving postoperative residual hearing.

In their study, Derinsu at al.^24^ evaluated the round window approach and found that complete preservation of residual hearing was achieved in 35.48% of patients. Using the cochleostomy approach in the majority of cases, the current study found a more positive outcome than Derinsu et al.’s^24^ study, with complete preservation achieved in 50% of patients.

The majority of cases in the current study did not have any reported intraoperative complications. This is a positive prognostic indicator for the preservation of residual hearing. Intraoperative complications could result in intracochlear damage, which in turn may lead to loss of residual hearing, thereby affecting the preservation of residual hearing. According to Clark et al. (1988) and Balkany et al. (1999) (as cited in Di Nardo et al.^8^), surgical complications affect anatomical structures following implantation. Kiefer et al.^7^ have cautioned that damage to the cochlea could be a result of acoustic trauma from bone drilling during surgery which is required for the cochleostomy.

In Di Nardo et al.’s.^8^ study it was found that the cochlea was not that sensitive to trauma caused by surgery. The current researcher holds that this should result in positive outcomes with regard to preservation of residual hearing. However, 22% of implantees in Di Nardo's study experienced a total loss of hearing postoperatively. The current study had a more favorable outcome than that of Di Nardo et al.’s.^8^ study in that only 8% lost their hearing completely, thus illustrating successful preservation of residual hearing in the vast majority of cases.

The authors are of the view that the positive outcome in the current study is due to the expertise of the surgeons together with improved electrode designs, as evidenced by the minimal intraoperative complications. Despite the fact that the majority of implantees underwent complete insertion of the electrode array, minimal surgical complications occurred and successful hearing preservation was achieved.

## Conclusion

Preservation of residual hearing following cochlear implantation was successfully achieved in 92% of participants – 42% partial and 50% complete, with 8% of recipients experiencing a complete loss postoperatively. Similar results were obtained regardless of which type of surgical technique was used – AOS or contour on stylet. The majority of surgeons utilized a cochleostomy approach which proved to be successful. Minimal intraoperative complications were reported, resulting in a positive outcome, indicative of successful surgical techniques and expertise of the surgeons, as well as improved electrode designs. Current findings are of clinical significance, making EAS a reality.

With the increasing number cochlear implants, the expanding criteria for implantation,^10^ the continuous increasing success rate of cochlear implants over the last two decades^7^ and the positive performance indicators of preoperative residual hearing,^2^ preservation of residual hearing has taken a central stage, and current findings support this.

## Conflicts of interest

The authors declare no conflicts of interest.
